# The impact of health beliefs and trust in health information sources on SARS-CoV-2 vaccine uptake

**DOI:** 10.3389/fpubh.2024.1340614

**Published:** 2024-03-15

**Authors:** Sami Hamdan Alzahrani

**Affiliations:** ^1^Family Medicine Department, Faculty of Medicine, King Abdulaziz University, Jeddah, Saudi Arabia; ^2^Health Promotion Center Research Group, Deanship of Scientific Research, King Abdulaziz University, Jeddah, Saudi Arabia

**Keywords:** trust, COVID-19, vaccine uptake, health communication, health belief

## Abstract

**Background:**

Health beliefs may mediate the relationship between trust and vaccination decisions, as confidence in online health information has expanded quickly. However, little is known about how health attitudes and trust in health information affect COVID-19 vaccine intention. This study aimed to assess the effect of health beliefs and trust in information sources on the willingness to receive a COVID-19 vaccine among the general public in Saudi Arabia.

**Methods:**

This study was designed and carried out at the Faculty of Medicine, King Abdulaziz University, Jeddah, Saudi Arabia. Selected items were extracted from the Saudi Residents’ Intention to Get Vaccinated Against COVID-19 (SRIGVAC) survey. They were categorized and validated into constructs of a health belief model (the perceived threat of COVID-19, vaccine-related benefits, barriers, and safety concerns) and trust in health information (from online platforms and health authorities/providers). Regression analysis and parallel mediation were used to assess the predictors of vaccination intentions.

**Results:**

Based on the responses of 3,091 participants, vaccine-related barriers and safety concerns negatively influenced vaccination intention, whereas vaccine benefits and the perceived threat of COVID-19 were positively correlated with vaccination intention. Trust in online health information had a direct relationship with intentions (β = 0.09, *p* < 0.0001) as well as indirect relationships through the perceived benefits (β = 0.095), the perceived barriers (β = −0.029), and the perceived safety concerns toward the vaccine (β = −0.010). The relationship between the willingness to vaccinate and trust in authentic information was fully mediated by all domains of health beliefs, with indirect coefficients of 0.004, 0.310, −0.134, and −0.031 for the perceived threat, vaccine benefits, barriers, and safety concerns, respectively.

**Conclusion:**

The relationship between the willingness to vaccinate and trust in authentic information was fully mediated by all domains of health beliefs. Vaccine coverage in Saudi Arabia can be optimized by targeting the health beliefs of the general public.

## Introduction

The coronavirus disease (COVID-19) has impacted the global public health sector and caused economic, mental, and social turmoil (1). Research and published literature have expanded significantly since the discovery of SARS-CoV-2 to better understand several aspects of the disease, such as transmission, pathophysiology, therapy, diagnostics, vaccine development and utilization, and people’s attitudes and misconceptions (2, 3).

There were 830,127 confirmed cases and 9,618 deaths, and 77.58% of the population received at least one dose till March 10, 2023 (4). The COVID-19 vaccination hesitancy rates in the United States range from 2.69 to 26.7% (5). A Saudi survey revealed that 36.9% of people hesitated to vaccinate (6). Behavioral determinants for COVID-19 vaccination are important because they determine whether people choose to be vaccinated or not. Understanding and managing these behavioral variables allows public health efforts to be more focused, resulting in increased vaccination acceptance and higher levels of population immunity, which is critical for preventing the development of infectious illnesses such as COVID-19. Understanding these determinants also allows public health officials, lawmakers, and healthcare professionals to develop effective vaccination promotion initiatives.

In Saudi Arabia, various trustworthy sources of information on COVID-19 exist, including the Saudi Ministry of Health (MOH), the Saudi Center for Disease Prevention and Control (Weqaya), the Saudi Press Agency, and local health agencies. The Saudi Ministry of Health’s official website featured COVID-19-related information, directives, and resources. It contains information about testing, vaccination, and health measures. The Weqaya platform offered COVID-19 information, such as statistics, guidelines, and resources. It offered updates on the situation in Saudi Arabia as well as preventive measures.

Several COVID-19 vaccines have been authorized and are now being used globally (7). Estimates indicate that approximately 60–70% of the general public should be vaccinated to attain herd immunity (8). A data-driven model of SARS-CoV-2 transmission suggested that vaccine-induced herd immunity would require coverage of 93% or higher because not all vaccines have equal efficacy and the emergence of new resistant variants (9). However, the behavioral intentions of individuals to get vaccinated are major determinants of the successful establishment of the threshold of herd immunity. These intentions can be impacted by concerns regarding the rapidity of vaccine development, the perceived barriers to vaccination, and the accumulated data from different sources of information that formulate individuals’ perceptions (10, 11).

For this reason, it is important to quantify the behavioral determinants of the general public’s desire to get the COVID-19 vaccine via reliable and validated measures. The health belief model (HBM) has been frequently used in the literature to measure the ability of people to make health-related decisions based on distinct variables, including the perceived susceptibility to and severity of a disease, the perceived benefits of engagement in a health-promoting behavior, the perceived barriers, and cues to actions (12). The HBM has been a useful tool for predicting short- and long-term health-related behaviors, and it has been recently validated in studies investigating the behavioral intentions to get the COVID-19 vaccine (13, 14). Nevertheless, scholars have revealed a limited power of the HBM measurements for behavioral prediction, and they suggested extending the variables of HBM models to improve their explanatory power (15, 16).

Concomitantly, focusing on the context of COVID-19, the behavioral intentions might have been affected by other factors that go beyond those utilized in the HBM model. For example, due to the lack of information about the vaccine, individuals may rely on a trusted party to make a risk/benefit-based decision to get the vaccine (17). In essence, the trusted party usually holds the best interests and the expected competence that would ultimately help reduce decision complexity by the individuals (18). Therefore, trust in health information from scientific/evidence-based sources, such as national and international health authorities and healthcare providers, might directly influence the levels of vaccine uptake. The COVID-19 pandemic witnessed a surge in the utilization of social media platforms to obtain information, which was concurrently linked to heightened levels of stress among the general populace (19). Moreover, vaccine acceptance is likely to be affected by the information retrieved from internet sources. This is because trust in health information obtained from online sources and social media has grown rapidly, with approximately 72–83% of individuals seeking medical information in the United States and Europe (20), and 33% of Saudi residents receiving health information from social media on daily and weekly basis (21). Rather than these direct effects, health beliefs may act as potential mediators that alter the relationship between trust and vaccination decisions. However, little is known about the impact and the explanatory power of health beliefs and trust in health information on the intention to get the COVID-19 vaccine, and the knowledge about such a domain in Saudi Arabia is no exception. In the present study, we have adapted multiple items from the questionnaire used in the Saudi Residents’ Intention to Get Vaccinated Against COVID-19 (SRIGVAC) study to investigate the role of health beliefs and trust in health information on vaccination intentions among the general public in Saudi Arabia.

## Methods

### Study design

The study data was derived from the SRIGVAC study (22). In brief, the SRIGVAC study employed a cross-sectional, survey-based design based on a 56-item questionnaire (the items are provided in the Supplementary material), which assessed participants’ intentions to receive the COVID-19 vaccine in addition to their personal perceptions about the potential benefits and harms of COVID-19 vaccination, the perceived barriers, and the perceived trust in the sources of information about the vaccine. The study was carried out between “December 25, 2020, and February 15, 2021.” All the samples were collected by an online questionnaire. The questionnaire and data collection details have been mentioned in the SRIGVAC study (22). The responses of 3,091 participants had been collected and were previously analyzed for the demographic predictors of the vaccination intent (22).

### Ethics statement

protocol of the present study was approved by the Research Ethics Committee (REC) of King Abdulaziz University, Jeddah, Saudi Arabia (Reference No. 422-23-11). Additionally, all experiments were performed in accordance with relevant guidelines and regulations (23–25). Written informed consent was obtained and documented from all participants. They were informed about the nature of the study and the confidentiality of their responses.

### Study instrument

Participants were requested to complete a structured online questionnaire distributed digitally via various social media channels (22). The first screen notified potential participants about the survey’s objectives and included an informed consent notification. The questionnaire was written in English, but most participants spoke Arabic. Thus, two bilingual translators handled the bidirectional translation. The questionnaire was then revised to increase respondents’ comprehension while retaining its content and meaning. A pilot test with fifty individuals from the general public was undertaken to ensure that the questionnaire was comprehensible, and it was then further modified as needed. The questionnaire’s reliability was 0.82 and measured by Cronbach’s alpha. Two senior faculty members and a medical educationist examined the questionnaire’s construct and content validity, and it was modified as recommended. The snowball technique was employed to acquire data due to COVID-19 constraints. The calculated sample size was 770, and we further inflated that number to get valid and generalizable results. The responses of 3,091 participants had been collected and were previously analyzed for the demographic predictors of vaccination intent (22).

### Measures

Initially, 35 items were selected based on the study’s objectives and conceptual framework. Of them, nine items were related to the demographic and clinical characteristics of the participants, including age, gender, educational level, nationality, current employment status, monthly income, household size, geographic location, and the status of receiving an influenza vaccine recently. Additionally, the participants’ behavioral intentions were assessed using a single item: “If the Covid-19 vaccine has become available in your country and it is recommended to you for free by the government, would you likely to receive it?.” The responses were graded on a five-point Likert scale, ranging from strongly disagree (1) to strongly agree (5) (Supplementary Table S1). A participant with a high score had a more positive intention to get vaccinated.

Subsequently, the remaining items (*n* = 25) were entered in an exploratory factor analysis (EFA) to classify the retrieved items into valid constructs (the analysis was performed in SPSS v.26, SPSS Inc., Chicago, IL, United States). Based on a Promax rotation method with an Eigenvalue of >1, the EFA indicated a six-factor solution (Kaiser-Meyer-Olkin Measure of Sampling Adequacy = 0.927, Chi-Square = 6164.21, *p* < 0.0001). The obtained items were categorized into the following domains and constructs: (1) the health beliefs domain, including the perceived threat of COVID-19 (*n* = 2), the perceived benefits of the SARS-CoV-2 vaccine (*n* = 5), the perceived barriers of the vaccine (*n* = 6), and the perceived safety concerns (*n* = 7); (2) the perceived trust domain, including the trust in online sources (*n* = 2) and health authorities and healthcare providers (*n* = 3) as sources of information about the vaccine. Notably, the perceived COVID-19 threat domain included a combination of the susceptibility and severity domains as described previously (26). Additionally, although the perceived barriers domain usually includes safety-related barriers, safety concerns were added to a separate construct to assess its direct effects on the primary outcomes exclusively and to explore its potential interaction with the trust domains on vaccination intentions (27). The obtained constructs from the EFA were further validated in a confirmatory factor analysis (AMOS V.26). The model showed acceptable goodness-of-fit statistics, with a significant chi-square value (χ^2^ = 2932.50, df = 293, *p* < 0.0001) and adequate indicators of fit indices (RMSEA = 0.054; CFI = 0.961; TLI = 0.953; GFI = 0.925). Therefore, no additional modifications were carried out. Table 1 shows the descriptive statistics, the response structure, the estimated standardized factor loadings of all items, and the outcomes of the reliability analysis for all domains.

**Table 1:** Descriptive and validity statistics of the study measures.

Study measurements	Scale	Mean ± SD	SFL	Cronbach’s alpha
Perceived threat of COVID-19				0.750
In your opinion, to what extent does the emerging corona virus, COVID-19, pose a threat to people in your country?	No risk (1)-Very high risk (4)	2.21 ± 0.89	0.887	
In your opinion, to what extent does the emerging corona virus, COVID-19, pose a threat to you?	No risk (1)-Very high risk (4)	1.80 ± 1.09	0.690	
Perceived benefits				0.847
Vaccines are effective in preventing the emerging corona	Strongly Disagree (1)-Strongly Agree (5)	3.43 ± 1.00	0.707	
The new Corona vaccines are safe	Strongly Disagree (1)-Strongly Agree (5)	3.38 ± 1.03	0.841	
The government should enforce everyone to get vaccinated	Strongly Disagree (1)-Strongly Agree (5)	2.87 ± 1.17	0.611	
Vaccines are a big advance for humanity	Strongly Disagree (1)-Strongly Agree (5)	3.93 ± 1.03	0.680	
To protect public health, we must follow government guidelines on vaccines	Strongly Disagree (1)-Strongly Agree (5)	4.05 ± 1.00	0.723	
Perceived barriers				0.938
I will refuse the vaccine because of the side effects	Strongly Disagree (1)-Strongly Agree (5)	2.94 ± 1.15	0.859	
I will refuse the vaccine because the clinical trials are done quickly	Strongly Disagree (1)-Strongly Agree (5)	3.09 ± 1.19	0.819	
I will refuse the vaccine because it will not be effective for preventing infection with the virus	Strongly Disagree (1)-Strongly Agree (5)	2.76 ± 1.12	0.902	
I will refuse the vaccine because the chances of me being at risk of contracting the emerging virus are low, so the vaccination is meaningless	Strongly Disagree (1)-Strongly Agree (5)	2.73 ± 1.16	0.830	
I will refuse the vaccine because the pandemic or vaccinations are a conspiracy of companies or organizations	Strongly Disagree (1)-Strongly Agree (5)	2.43 ± 1.16	0.800	
I will reject the vaccine because the vaccinations represent a trick by the pharmaceutical companies and the organizations that promote them for financial gain	Strongly Disagree (1)-Strongly Agree (5)	2.48 ± 1.18	0.808	
Perceived safety concerns				0.957
Corona vaccines contain mercury in dangerous quantities	Strongly Disagree (1)-Strongly Agree (5)	2.64 ± 0.97	0.773	
Corona vaccines contain dangerous ingredients	Strongly Disagree (1)-Strongly Agree (5)	2.63 ± 1.05	0.831	
Corona vaccines cause autism	Strongly Disagree (1)-Strongly Agree (5)	2.39 ± 1.03	0.873	
Corona vaccines cause infertility in women	Strongly Disagree (1)-Strongly Agree (5)	2.49 ± 1.00	0.902	
Corona vaccines cause infertility in men	Strongly Disagree (1)-Strongly Agree (5)	2.50 ± 0.99	0.896	
Corona vaccines cause AIDS	Strongly Disagree (1)-Strongly Agree (5)	2.24 ± 0.99	0.878	
Corona vaccines cause death	Strongly Disagree (1)-Strongly Agree (5)	2.46 ± 1.05	0.887	
Perceived trust (online sources)				0.789
Evaluate your reliability regarding the information on the new Corona COVID-19 vaccines: websites	Very Unconfident (1)- Very Confident (5)	3.04 ± 1.07	0.820	
Evaluate your reliability regarding the information on the new Corona COVID-19 vaccines: social media applications	Very Unconfident (1)- Very Confident (5)	2.87 ± 1.12	0.795	
Perceived trust (health authorities)				0.791
Evaluate your reliability regarding the information on the new Corona COVID-19 vaccines: Ministry of Health	Very Unconfident (1)- Very Confident (5)	4.29 ± 0.94	0.893	
Evaluate your reliability regarding the information on the new Corona COVID-19 vaccines: WHO	Very Unconfident (1)- Very Confident (5)	3.65 ± 1.20	0.644	
Evaluate your reliability regarding the information on the new Corona COVID-19 vaccines: healthcare providers	Very Unconfident (1)- Very Confident (5)	3.94 ± 0.93	0.764	

SFL, standardized factor loadings.

### Statistical analysis

SPSS v.26 was used to conduct the statistical analysis. The mean scores of different items, including the variables of health beliefs and trust as well as the intention to get vaccinated scale, were calculated based on the overall mean value of each item. Bivariate associations between the continuous variables were investigated using Pearson’s correlation, and the results were presented in a correlation matrix. The univariate associations between participants’ intention to get vaccinated (as a continuous variable) and demographic characteristics were assessed using *t* tests (for gender, nationality, and the previous history of receiving an influenza vaccine) and one-way analysis of variance (ANOVA) for other demographic variables. A three-step hierarchical linear multiple regression analysis was employed to explore the predictors of vaccination intention, where the significantly associated factors from the univariate correlation tests were exclusively included as independent variables and the intention to receive the vaccine was the dependent variable. Demographic variables were entered in Block 1, health beliefs variables in Block 2, and trust in information sources in Block 3. Such an approach was used to test whether individuals’ trust could influence the likelihood of getting vaccinated against COVID-19, above and beyond demographic characteristics and health beliefs. The results of the regression model were expressed as standardized regression coefficients (β) and their respective 95% confidence intervals (95%CIs). The amount of variance explained in the model as well as the changes in the amount of variance were presented as R2 and changes in R2, respectively. A *p*-value of < 0.05 indicated statistical significance. Therefore, to assess whether distinct health beliefs have accounted for such relationships, we carried out a parallel mediation analysis using the PROCESS macro in SPSS (28). Such an analysis considers multiple dimensions as potential mediators while accounting for the shared variance between them (28).

## Results

In the domain of perceived threat of COVID-19, participants perceive a moderate threat of COVID-19 to people in their country (mean = 2.21, SD = 0.89), while participants perceive a lower threat to themselves (mean = 1.80, SD = 1.09). The internal consistency reliability (Cronbach’s alpha) for the perceived threat scale was good (0.770). In the domain of perceived barriers, participants express some concerns or potential barriers to vaccination, such as side effects and skepticism about clinical trials. The mean scores for perceived barriers range from 2.43 to 3.09. This section showed high internal consistency with a Cronbach’s alpha value of 0.938. In the domain of perceived safety concerns, participants disagree with various misinformation regarding vaccine safety (e.g., mercury content, causing autism or infertility). The mean scores for safety concerns range from 2.24 to 2.64. Internal consistency was good, with a Cronbach’s alpha value of 0.957. Other domain scores are shown in Table 1.

Regarding the primary outcome variable, when the participants were asked about their intentions to get the COVID-19 vaccine, 9.8% of the participants responded as “strongly disagree,” 10.5% as “disagree,” 26.8% as “neither agree nor disagree,” 23.2% as “agree,” and 29.7% as “strongly agree.” The mean ± SD intention score was 3.52 ± 1.28. Univariate analyses showed that the intent to receive the vaccine was significantly higher among males (*p* < 0.001), Saudis (*p* < 0.001), as well as the participants with < secondary education (*p* = 0.001), a monthly income of SAR10,000 or higher (*p* = 0.002), and those residing in the Southern region (*p* < 0.001) compared to their peers. In addition, respondents who had received an influenza vaccine were significantly more likely to be willing to get vaccinated than those who had not received the vaccine (*p* < 0.001, Table 2).

**Table 2:** Demographic differences in the intention to get the COVID-19 vaccine.

Parameter	Category	Mean	SD	*p*-value
Age	18–29 y	3.56	1.27	0.066
	30–44 y	3.46	1.31	
	45–59 y	3.59	1.25	
	≥60 y	3.83	1.05	
Gender	Female	3.32	1.31	<0.001*
	Male	3.84	1.16	
Nationality	Saudi	3.56	1.27	<0.001*
	Non-Saudi	3.24	1.36	
Educational level	<Secondary education	4.21	0.93	0.001*
	Secondary	3.37	1.31	
	University	3.53	1.28	
	Post-graduate	3.53	1.28	
Employment status	Employed-Government	3.57	1.26	0.388
	Private/self-employed	3.51	1.34	
	Student	3.54	1.26	
	Not working	3.46	1.29	
Monthly income (SAR)	<3,000	3.51	1.28	0.002*
	3,000–10,000	3.43	1.32	
	>10,000–25,000	3.60	1.24	
	>25,000	3.86	1.20	
Household size	1–3	3.49	1.29	0.055
	4–6	3.50	1.27	
	7–9	3.52	1.29	
	≥10	3.71	1.30	
Geographic location	Western	3.53	1.25	<0.001*
	Eastern	3.45	1.33	
	Northern	3.38	1.30	
	Central	3.37	1.36	
	Southern	3.88	1.18	
Received an influenza vaccine shot in the past year	Yes	3.87	1.17	<0.001*
	No	3.28	1.30	

*p-value of < 0.05 indicated statistical significance.

Table 3 shows the relationship between participants’ intention to receive the COVID-19 vaccine and the items of HBM and trust in information sources. Intention to get a COVID-19 vaccine was positively correlated with the perceived threat of COVID-19 and the perceived benefits of the vaccine, while it was negatively correlated with the perceived barriers and the perceived safety concerns toward the vaccine. In addition, willingness to get the vaccine was positively associated with the perceived trust in online information sources and information obtained from health authorities/healthcare providers (Table 3).

**Table 3:** Correlation matrix for the relationships between COVID-19 vaccine uptake and the variables of the health beliefs model and trust in information sources.

Variable	1	2	3	4	5	6
1. Intention to get the vaccine						
2. Perceived threat of COVID-19	0.118**					
3. Perceived benefits	0.703**	0.083**				
4. Perceived barriers	−0.573**	−0.093**	−0.553**			
5. Perceived safety concerns	−0.408**	−0.016	−0.459**	0.701**		
6. Perceived trust in online sources	0.138**	−0.021	0.163**	0.075**	0.118**	
7. Perceived trust in health authorities or healthcare providers	0.431**	0.095**	0.549**	−0.402**	−0.363**	0.202**

*p-value of < 0.05 indicated statistical significance.

The significantly associated categorical and continuous variables with the intention to receive the COVID-19 vaccine (Tables 2, 3) were further entered in hierarchical multivariate regression models to test the independent predictors of the intent to vaccinate (Table 4). The control model containing the demographic variables (Model 1) explained 9.4% of the variation in vaccination intention, which increased with the addition of trust variables (29.1% for Model 2) and health beliefs variables (58.4% for Model 3).

**Table 4:** The results of the hierarchical regression analysis for the predictor of vaccination intentions among the general public in Saudi Arabia.

Parameter	Estimate	Model 1		Model 2		Model 3	
		Beta (95% CI)	*p*-value	Beta (95% CI)	*p*-value	Beta (95% CI)	*p*-value
	Adjusted R2	0.092		0.291		0.584	
	ΔR2	0.094		0.199		0.293	
Gender	Male	0.53(0.44 to 0.63)	<0.0001	0.46(0.38 to 0.55)	<0.0001	0.24(0.18 to 0.31)	<0.001*
	Female	Ref		Ref		Ref	
Nationality	Saudi	0.34(0.19 to 0.48)	<0.0001	0.29(0.16 to 0.42)	0.086	0.10(0.05 to 0.20)	0.048*
	Non-Saudi	Ref		Ref		Ref	
Educational level	<Secondary education	0.65(0.26 to 1.03)	0.001	0.40(0.06 to 0.74)	0.022	0.54(0.28 to 0.81)	<0.001*
	Secondary	−0.12(−0.30 to 0.06)	0.184	−0.09(−0.25 to 0.07)	0.272	0.10(−0.03 to 0.22)	0.126
	University	0.03(−0.09 to 0.15)	0.655	0.02(−0.09 to 0.13)	0.732	0.07(−0.02 to 0.15)	0.113
	Post-graduate	Ref		Ref		Ref	
Monthly income (SAR)	<3,000	−0.03(−0.28 to 0.22)	0.795	−0.09(−0.31 to 0.13)	0.405	0.001(−0.17 to 0.17)	0.973
	3,000–10,000	−0.26(−0.51 to −0.02)	0.037	−0.25(−0.47 to −0.03)	0.026	−0.11(−0.28 to 0.06)	0.188
	>10,000–25,000	−0.28(−0.51 to −0.04)	0.025	−0.22(−0.44 to −0.01)	0.038	−0.09(−0.25 to 0.07)	0.276
	>25,000	Ref		Ref		Ref	
Geographic location	Western	−0.25(−0.40 to −0.11)	0.001	−0.20(−0.33 to −0.07)	0.002	−0.18(−0.27 to −0.08)	<0.001*
	Eastern	−0.32(−0.51 to −0.12)	0.001	−0.23(−0.40 to −0.06)	0.008	−0.18(−0.31 to −0.05)	0.008*
	Northern	−0.34(−0.56 to −0.12)	0.002	−0.32(−0.51 to −0.12)	0.001	−0.21(−0.36 to −0.06)	0.005*
	Central	−0.35(−0.52 to −0.18)	<0.0001	−0.24(−0.39 to −0.09)	0.002	−0.18(−0.30 to −0.06)	0.003*
	Southern	Ref		Ref		Ref	
Recently received an influenza vaccine	Yes	0.54(0.63 to 0.45)	<0.0001	0.41(0.49 to 0.33)	<0.0001	0.19(0.26 to 0.13)	<0.001*
	No	Ref		Ref		Ref	
Trust in information sources	Trust in information from online sources	NA	NA	0.06(0.02 to 0.1)	0.004	0.09(0.06 to 0.12)	<0.001*
Trust in information sources	Trust in information from health authorities	NA	NA	0.64(0.59 to 0.68)	<0.0001	0.01(−0.03 to 0.06)	0.633
HBM	Perceived threat of COVID-19	NA	NA	NA	NA	0.04(0.01 to 0.08)	0.014*
HBM	Perceived benefits	NA	NA	NA	NA	0.79(0.73 to 0.84)	<0.001*
HBM	Perceived barriers	NA	NA	NA	NA	−0.43(−0.47 to −0.38)	<0.001*
HBM	Perceived safety concerns	NA	NA	NA	NA	−0.10(−0.05 to −0.15)	<0.001*

*p-value of < 0.05 indicated statistical significance. HBM, health belief model.

Regarding trust variables, vaccination intention was predicted by individuals’ trust in health information sources from online platforms (β = 0.06, *p* = 0.004) and authentic sources (β = 0.64, *p* < 0.001, Model 2). However, with the addition of health beliefs variables to the model (Model 3), the willingness to vaccinate was independently associated with trust in online information (β = 0.09, *p* < 0.001) but not with trust in authentic health information (Table 4).

**Figure 1:**
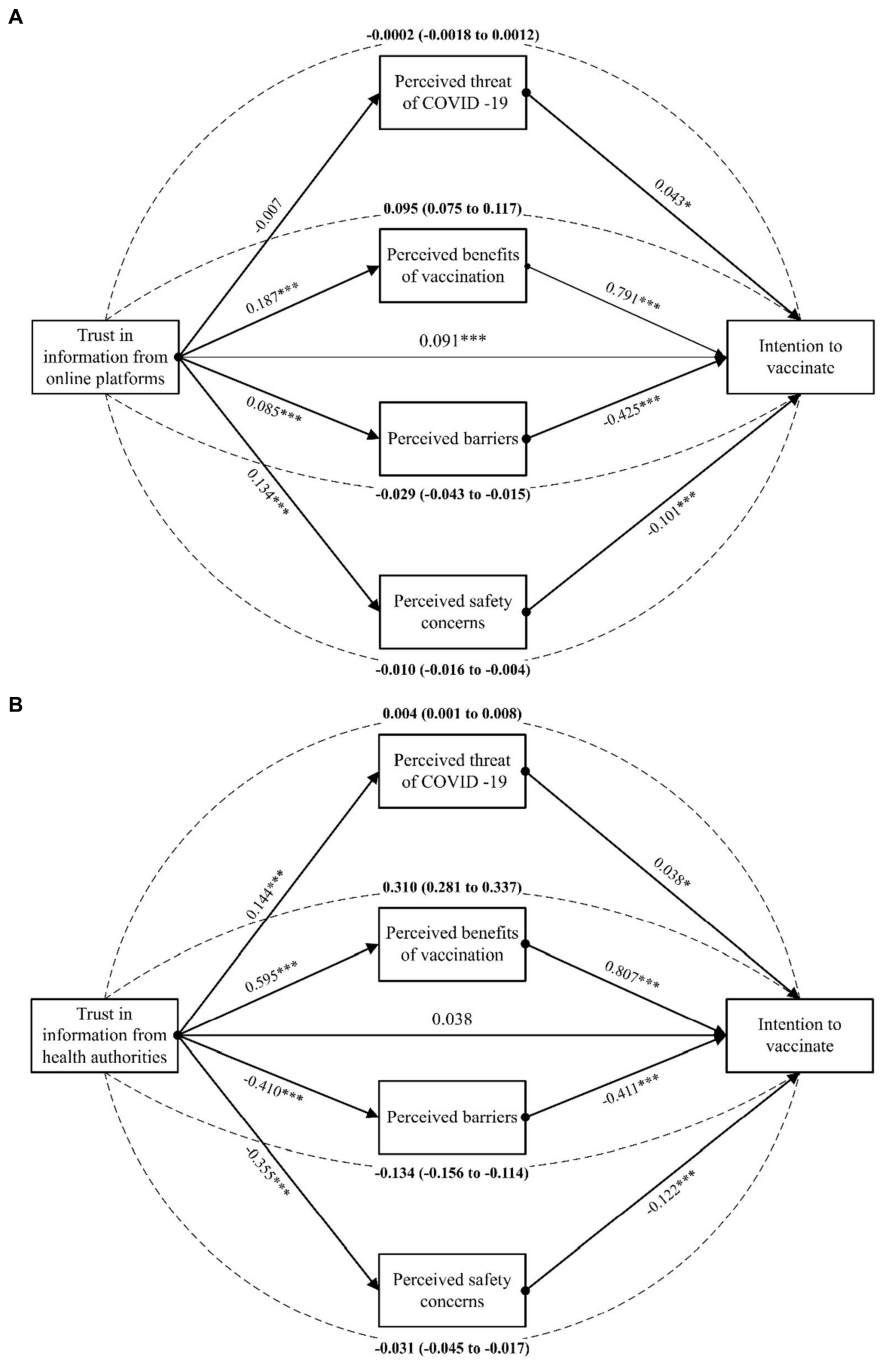
The results of the health beliefs as potential mediators of the relationship between vaccination intention and trust in health information from online sources **(A)** and from authentic sources **(B)**. Dashed lines indicate completely standardized indirect effects. *p < 0.05; **p < 0.01; ***p < 0.0001.

These results indicate that health beliefs have partially mediated the relationship between trust in online sources and vaccination intentions, and fully mediated the relationship between trust in authentic sources and vaccination intentions. Two separate mediation models were conducted, where each variable of trust in information sources was entered as a predictor variable in each model, health beliefs variables as parallel mediators, vaccination intention as a dependent variable, and demographic predictors as covariates (Figure 1). Based on a 95% bias-corrected confidence interval of 1,000 bootstrap samples, the indirect effect of trust in online information through the perceived benefits of the vaccine was entirely above zero (β = 0.095, 95% CI, 0.075 to 0.117), while the indirect effects through the perceived barriers and the perceived safety concerns were below zero (β = −0.029, 95%CI, −0.043 to −0.015 and β = −0.010, 95%CI, −0.016 to −0.004, respectively, Figure 1A). Additionally, the indirect coefficients for the relationship between trust in authentic information and vaccination intention were significant via all domains of the HBM, including the perceived threat of COVID-19 (β = 0.004, 95%CI, 0.001 to 0.008), the perceived benefits of the vaccine (β = 0.310, 95%CI, 0.281 to 0.337), the perceived barriers to vaccination (β = −0.134, 95%CI, −0.156 to −0.114), and the perceived safety concerns (β = −0.031, 95%CI, −0.045 to −0.017, Figure 1B).

## Discussion

Understanding the predictors of vaccine uptake is crucial to determine the reasons for vaccine hesitancy and promote vaccine coverage. Our results indicated that 52.9% of adults in the general public intend to receive the COVID-19 vaccine, which is lower than the required threshold for achieving herd immunity (8). As such, it is important to assess the independent personal health beliefs associated with vaccine acceptance and the main trusted parties that could communicate vaccine-related information as perceived by the general public. The results of the present study showed that health beliefs and trust in online information were independent predictors of vaccine acceptance. All the domains of HBM have fully mediated the relationship between vaccine uptake and trust in authentic health information (retrieved from healthcare providers, the Ministry of Health, or international health organizations), whereas vaccine-related health beliefs (vaccine benefits, barriers, and safety concerns) have partially mediated the impact of individuals’ trust in online information on vaccine acceptance.

Our findings are consistent with previous reports, which showed significant effects of the HBM constructs on vaccine uptake. For example, risk perception of COVID-19 was independently associated with the willingness to get vaccinated in Asia (29, 30), Europe (29), and the United States (31). Vaccine-related constructs, including the benefits, barriers, and safety concerns, were all significant factors that could explain vaccine uptake behavior in multiple investigations (29, 30, 32). A Chinese study reported “perceived benefits, cues to action, and various occupations” were positively associated with “vaccine acceptance.” In contrast, “perceived susceptibility and perceived barriers” were negatively associated with vaccine acceptance (33). These findings imply that public health authorities should communicate vaccine-related information based on the available clinical trials to fill the gap in the knowledge regarding vaccine efficacy and safety. However, targeting the perceived beliefs could be further optimized by getting deeper insights into the most trusted parties through which information could be communicated.

Generally, the core elements of trust include trust in the product itself (the vaccine), the provider (healthcare professionals), or the policy maker (the government or healthcare authorities) (34). In our study, since the vaccine was introduced during data collection, the trust in healthcare providers and policymakers may be exclusively meaningful. These sources are expected to communicate evidence-based information via reliable platforms, and the participants expressed the highest levels of trust in the Ministry of Health, followed by healthcare providers. However, although trust in national and international health authorities, as well as healthcare providers, was associated with the willingness to get vaccinated against COVID-19 in the univariate analysis, such a relationship was fully mediated by the health beliefs of individuals. In other words, trust in evidence-based information was associated with the intention to receive the vaccine, which was higher as mediated by the perceived threat of COVID-19 and the perceived benefits of vaccination and lower as mediated by the high levels of perceived barriers and safety concerns. Interestingly, a recent study observed that vaccine hesitancy had a negative relationship with age, family income, education status, coronavirus risk perception, faith in government, scientific and medical authorities, and traditional media, and was positively correlated with female gender, non-white ethnicity, and social media (35). Distrust, fear, and disinformation are important influencers of health beliefs about vaccination (36). Rathje et al. demonstrated that social media engagement is linked to vaccine views and that low-quality news sites predicted lower trust in the COVID-19 vaccine (37). Influencers can substantially impact COVID-19 vaccination uptake by building trust and distributing factual information or instilling fear, disinformation, and distrust. Public health campaigns should work strategically with influencers to increase positive influence and address concerns that may contribute to vaccine reluctance.

On the other hand, there was a significant direct effect of online platforms as sources of vaccine-related information on vaccination intention, irrespective of individual health beliefs. Moreover, the relationship between trust in online platforms and vaccine intention was relatively strengthened by higher perceived benefits and weakened by increased barriers and safety concerns, as reported by the participants. Similarly, Allington et al. have recently shown that the reliance on social media among US and UK residents was significantly greater than informational reliance on legacy media, which has finally impacted vaccine intentions (38). In addition, the use of social media to organize offline behavioral decisions in the UK has been associated with negative attitudes toward vaccination (39). This might underline the role of social media and health websites as external levers of vaccination decision-making.

The aforementioned results indicate that the trust in official, reliable sources of information was exclusively dependent on the personal beliefs. It is likely that people with unfavorable health beliefs toward the vaccine have been affected by other influencers that may actively oppose vaccination despite their perceived trust in health authorities. The present study showed several influencers, including the perceived threat of COVID-19, perceived benefits, perceived barriers, perceived safety concerns, perceived trust (online sources), and perceived trust (health authorities). Like the present study findings, Bateman et al. also mentioned influencers such as “perceived susceptibility, perceived severity, perceived obstacles, and others” in the constructs of the HBM (36).

Influencers are essential in creating perceptions of COVID-19 vaccination uptake, influencing people’s perspectives in various ways. Influencers who effectively convey perceived benefits might substantially impact vaccination acceptance by emphasizing the advantages of immunization in reducing disease and promoting community health. In contrast, influencers may contribute to perceived barriers by emphasizing possible adverse effects or uncertainties associated with the vaccine, causing public skepticism. Addressing perceived safety concerns is critical; influencers focusing on and communicating vaccines’ rigorous testing and monitoring processes can reduce anxiety and boost confidence. If influencers share reliable and evidence-based information, the perceived trust in online sources can positively affect opinions. However, the use of online platforms to disseminate misinformation may undermine confidence. Additionally, influencers can substantially impact perceived trust in health authorities by either approving or questioning their advice. Collaboration with influencers who agree with health officials’ statements can boost trust, whereas discordant messages can weaken public confidence in immunization efforts. Overall, influencers have a multidimensional impact on COVID-19 vaccination uptake by altering perceptions of advantages, barriers, safety, and trust in online sources and health authorities.

Notwithstanding the positive relationship between trust in online information and in vaccination intention, the role of online misinformation on vaccine hesitancy should not be neglected. It is recommended that public health beliefs should be targeted via well-organized, web-based strategies. First, social media companies should direct their users away from unreliable, low-quality information sources, and these should be replaced by trusted data from reputable content producers. Second, health authorities should transparently communicate evidence-based information preferentially via official social media pages and dedicated health websites that promote individuals’ trust in information and help in decision-making. Third, although social media platforms and online resources can be useful for disseminating information about vaccine safety and acceptance, a holistic approach is necessary for effective communication and education. This includes community engagement, peer-to-peer advocacy, health literacy initiatives, mobile health (mHealth) applications, and programs offered in schools, colleges, and workplaces.

### Strengths and limitations

The current study addresses a highly relevant and urgent problem by considering the factors influencing COVID-19 vaccine intention in Saudi Arabia. Given the global importance of pandemic immunization, the findings may provide significant insights. Including a large sample size (3,091) improves the statistical power and generalizability of the findings, making them more robust and credible. The study employs the HBM to categorize and validate selected items. This model is a well-established framework in health psychology, strengthening the study’s theoretical basis. The study recognizes the role of trust in online health information, which is particularly relevant in the era of information technology. Understanding the impact of online sources on vaccine intentions helps provide more comprehensive knowledge of health decision-making. We used a unique approach to examine the predictors of vaccination intentions. Rather than focusing on rational calculations of health beliefs, we extended the hypothetical framework of behavioral intentions to the social context that may shape those beliefs, aka trust in information sources. The study’s conclusion has practical implications for increasing vaccine coverage in Saudi Arabia, underscoring the significance of tackling health attitudes. This information is helpful for public health officials and policymakers tasked with developing effective immunization campaigns.

However, the cross-sectional nature of data collection might limit to obtain reliable causal relationships between the predictors and the outcome. Despite being the largest national study to date, the present study’s findings could not be generalized to other countries with different ethnic and cultural determinants of vaccination uptake. Finally, the online survey might have induced selection bias, where participants with active internet connections could access the survey. Finally, potential social desirability biases among participants could be a significant constraint. It is likely that participants provided social desirability bias by responding in ways that aligned with societal standards or were viewed as socially acceptable. This could affect the accuracy of stated attitudes and intentions.

## Conclusion

All HBM constructs were significant predictors of vaccination intentions; vaccine-related benefits and the perceived threat of COVID-19 were positively correlated, whereas vaccine barriers and safety concerns were negatively correlated. Trust in health websites and social media platforms was independently associated with the willingness to vaccinate, and it was partially mediated by HBM variables. Trust in authentic information from governmental organizations and healthcare providers was fully mediated by HBM constructs. The present study highlights the significance of online platforms on vaccine uptake, whereas the role of information from authentic sources (the government, healthcare providers, etc.) was exclusively dependent on the health beliefs of individuals. Utilizing the general public’s health beliefs can improve vaccine coverage in Saudi Arabia.

## Data availability statement

The original contributions presented in the study are included in the article/Supplementary material, further inquiries can be directed to the corresponding author.

## Ethics statement

The studies involving humans were approved by the protocol of the present study was approved by the Research Ethics Committee (REC) of King Abdulaziz University, Jeddah, Saudi Arabia (Reference No. 422-23-11). The studies were conducted in accordance with the local legislation and institutional requirements. The participants provided their written informed consent to participate in this study.

## Author contributions

SA: Writing – review & editing, Writing – original draft, Resources, Project administration, Methodology, Investigation, Funding acquisition, Formal analysis, Data curation, Conceptualization.
