# Supplementary material for: The impact of health beliefs and trust in health information sources on SARS-CoV-2 vaccine uptake
Source: Front Public Health. 2024 Mar 15;12:1340614. doi: 10.3389/fpubh.2024.1340614 (PMC10978693; doi:10.3389/fpubh.2024.1340614)
Supplement: Supplementary file 1 [file Table_1.DOCX]

Table S1: The used items and domains in the current study.

| **Demographic characteristics** | Age |
| --- | --- |
|  | Gender |
|  | How many people do you live with in the same house, including yourself? |
|  | what is your original country? |
|  | What is your current residence? |
|  | What is your educational level? |
|  | What is your employment status now? |
|  | Are you currently working completely from your home? |
|  | Do you work partially from home (remotely) or have your working hours been reduced due to the current Corona Covid 19 pandemic? |
|  | Do you work in any of the specialties related to health care? |
|  | How much is your average monthly income? (SAR) |
| **Clinical history** | Do you have any chronic disease (such as: diabetes - pressure - asthma - heart or kidney disease - immune diseases - obesity)? |
|  | Have you been infected with the new Corona Covid 19 virus? |
|  | Has any of your family members been infected with the new emerging corona virus, Covid 19? |
|  | Has any of your friends or co-workers been infected with the emerging corona virus, Covid 19? |
|  | Have you ever been vaccinated against seasonal influenza during this year or the previous year? |
| **Knowledge and perceptions about COVID-19** | How would you rate your level of knowledge about the emerging corona virus, Covid 19? |
|  | In your opinion, to what extent does the emerging corona virus, Covid 19, pose a threat to people in your country? |
|  | In your opinion, to what extent does the emerging corona virus, Covid 19, pose a threat to you? |
| **Attitudes** | If the Covid-19 vaccine became available in your country and was recommended to you for free by the government, would you take it? |
|  | If the vaccine is not free and recommended for you by the health authorities, will you pay money to take it? |
|  | If you are not from those how will take the vaccination in the first free stages, will you look to buy the vaccination to take it early? |
|  | If you are among the categories eligible to receive the new Corona Covid 19 vaccine, will you take it immediately and at the earliest opportunity? |
|  | If you have children under 16 years old, do you accept to be vaccinated with the new Corona Covid 19 vaccine? |
| **Attitudes towards vaccine safety** | Corona vaccines contain mercury in dangerous quantities |
|  | Corona vaccines contain dangerous ingredients |
|  | Corona vaccines cause autism |
|  | Corona vaccines cause infertility in women |
|  | Corona vaccines cause infertility in men |
|  | Corona vaccines cause AIDS |
|  | Corona vaccines cause death |
| **Attitudes towards vaccine efficacy** | Vaccines are effective in preventing the emerging corona. |
|  | The new Corona vaccines are safe |
|  | The government should inforce everyone to get vaccinated |
|  | Vaccines are a big advance for humanity |
|  | To protect public health, we must follow government guidelines on vaccines. |
|  | Vaccines only prevent severe infection. |
| **Reasons for refusal** | I will refuse the vaccine because of the side effects |
|  | I will refuse the vaccine because the clinical trials are done quickly |
|  | I will refuse the vaccine because it will not be effective for preventing infection with the virus |
|  | I will refuse the vaccine because the chances of me being at risk of contracting the emerging virus are low, so the vaccination is meaningless |
|  | I will refuse the vaccine due to my lack of confidence in the health and scientific authorities |
|  | I will refuse the vaccine because the pandemic or vaccinations are a conspiracy of companies or organizations |
|  | I will reject the vaccine because the vaccinations represent a trick by the pharmaceutical companies and the organizations that promote them for financial gain |
|  | Vaccines are not necessary because they target a relatively harmless disease. |
|  | Corona infection provides better immunity than vaccines. (Injury and natural recovery are a better option for acquiring immunity) |
|  | Vaccines contradict my belief that I must use natural products and avoid toxins. |
|  | I am morally opposed to getting vaccinated |
| **Trust in the sources of information** | Evaluate your reliability regarding the information on the new Corona Covid 19 vaccines: the television |
|  | Evaluate your reliability regarding the information on the new Corona Covid 19 vaccines: newspapers and magazines |
|  | Evaluate your reliability regarding the information on the new Corona Covid 19 vaccines: websites |
|  | Evaluate your reliability regarding the information on the new Corona Covid 19 vaccines: Social media applications |
|  | Evaluate your reliability regarding the information on the new Corona Covid 19 vaccines: Friends or family |
|  | Evaluate your reliability regarding the information on the new Corona Covid 19 vaccines: Healthcare providers |
|  | Evaluate your reliability regarding the information on the new Corona Covid 19 vaccines: Ministry of Health |
|  | Evaluate your reliability regarding the information on the new Corona Covid 19 vaccines: WHO |
